# Dietary medium chain triglycerides impairs orexigenic action of ghrelin in mice

**DOI:** 10.3389/fendo.2025.1690761

**Published:** 2026-01-05

**Authors:** Daisuke Aotani, Hiroyuki Ariyasu, Tomohiro Tanaka, Satoko Shimazu-Kuwahara, Hidenari Nomura, Yoshiyuki Shimizu, Katsushi Takeda, Hiroyuki Koyama, Toru Kusakabe, Takashi Miyazawa, Takatoshi Hikida, Hiromi Kataoka, Kazuwa Nakao

**Affiliations:** 1 Medical Innovation Center, Kyoto University Graduate School of Medicine, Kyoto, Japan; 2 Department of Gastroenterology and Metabolism, Nagoya City University, Graduate School of Medical Sciences, Nagoya, Japan; 3 Center for Diabetes, Endocrinology and Metabolism, Shizuoka General Hospital, Shizuoka, Japan; 4 Department of Diabetes, Endocrinology and Nutrition, Kyoto University Graduate School of Medicine, Kyoto, Japan

**Keywords:** ghrelin, medium-chain triglycerides, food intake, hormone signaling, growth hormone

## Abstract

**Introduction:**

Ghrelin, a stomach-derived hormone, increases food intake and body weight. Efforts have been made therefore to modulate ghrelin signaling for the treatment of obesity or emaciation. However, basic biology of the potential effects of dietary nutrients on ghrelin action has not yet been fully uncovered.

**Methods:**

To investigate an impact of fat intake on orexigenic effect of ghrelin, we examined ghrelin transgenic mice or mice treated with ghrelin fed with the diet containing medium-chain triglycerides (MCT).

**Results:**

Five-day MCT diet feeding increased plasma ghrelin levels by 2.5-fold compared with long-chain triglyceride (LCT)-fed mice, potentially through *O*-acyltransferase-mediated medium-chain fatty acylation and maturation of ghrelin. The plasma ghrelin levels of ghrelin and *O*-acyltransferase double transgenic mice (ghrelin-Tg) reached ten times higher than wild-type (WT) littermates under the MCT diet. The rise of plasma ghrelin levels in ghrelin-Tg, however, was not associated with any changes in food intake or body weight. Administration of ghrelin significantly increased food intake in WT mice under the normal chow (NC) or the LCT diet. In contrast, ghrelin-induced increase of food intake was not observed under the MCT diet. Consistently, upregulation of hypothalamic expression of neuropeptide Y (NPY), a critical mediator of orexigenic action by ghrelin, was observed under the NC or the LCT diet, but not under the MCT diet. Meanwhile, enhancement of food intake by the intracerebroventricular injection of NPY was preserved in mice fed with the MCT diet, suggesting an interference of ghrelin signaling upstream of NPY. Interestingly, ghrelin-induced growth hormone (GH) secretion was not attenuated by the MCT diet, indicating a distinct pathway for appetite and GH regulation.

**Discussion:**

Our results provide evidence for the MCT-induced attenuation of the orexigenic effect of ghrelin, and suggest a novel interplay between dietary lipids and hormone signaling.

## 1 Introduction

Ghrelin was first discovered as an endogenous agonist of the growth hormone (GH) secretagogue receptor (GHSR) (1). Following studies have revealed its critical role in energy homeostasis as an enhancer of hunger, food intake, and adiposity (2–5). Based on expanding knowledge of physiologic roles of ghrelin, compounds that modulate ghrelin production or function have extensively been studied for the development of drugs to treat nutrition-related morbidities such as obesity, emaciation, or cachexia.

A unique post-translational modification by octanoate at the third Serine (Ser^3^) residue of ghrelin peptide by ghrelin *O*-acyltransferase (GOAT) is essential for its biological action (6, 7). Octanoate or C8:0 is a member of saturated medium-chain fatty acids (MCFA) found in dietary lipids such as coconut oil. MCFA is an edible oil that typically comprises octanoate (caprylic acid) and decanoate (capric acid). MCFA displays biophysical and metabolic properties distinct from long-chain fatty acids (LCFA); MCFA is absorbed through the portal vein instead of the lymphatic vessels, is not re-esterified to triacylglycerol within intestinal cells like in the case of LCFA, and is more rapidly beta-oxidized in the liver (8, 9).

Former studies have found that the ingestion of the diet containing MCFA including octanoate leads to an enhanced synthesis and secretion of mature octanoylated ghrelin *in vivo* (10–12). The discovery of ghrelin acylation by MCFA has opened up a new era for the study of nutritional biology of dietary medium-chain triglycerides (MCT).

Accumulating evidence has recently provoked the notion that dietary nutrients interact with the production or the function of hormones potentially through its modulatory effects on hormone receptor signaling, leading to the hypothesis of nutrient-endocrine crosstalk. One of the best examples is the case of leptin, where leptin signaling is well known to be impaired by an excess of LCFA in the diet (13). Ingestion of LCFA has also been reported to decrease ghrelin production (14, 15) and inhibit its hyperphagic effect in mice (16). Besides ghrelin production or secretion, potential effects of dietary MCFA on ghrelin function still need to be clarified.

In the present study, we sought to determine the effect of dietary MCT on ghrelin action, focusing on its orexigenic effect *in vivo*.

## 2 Materials and methods

### 2.1 Animal experiments

Male C57BL6/J mice were purchased from Japan SLC (Shizuoka, Japan). Mice were housed individually under a 12/12 h light/dark cycle (lights on at 08.00 h), at a constant room temperature of 24 ± 1 °C and with free access to food and water unless otherwise indicated. All experiments were performed with 9-10-week-old mice.

Generation of des-acyl ghrelin transgenic mice has been described previously (17). In these mice, des-acyl ghrelin, which lacks the n-octanoyl modification at Ser^3^ of the ghrelin protein, is overexpressed in the liver with elevated plasma level. Similar to the case of des-acyl ghrelin transgenic mice, GOAT transgenic mice were generated by a fusion gene of the human serum-amyloid-P promotor and mouse GOAT cDNA full length coding sequences. Ghrelin transgenic (ghrelin-Tg) mice were generated by mating des-acyl ghrelin transgenic mice and GOAT transgenic mice, in which both des-acyl ghrelin and GOAT are overexpressed. Male mice with des-acyl ghrelin Tg/+: GOAT Tg/+ genotype were used as ghrelin-Tg for the experiments. Wild-type (WT) littermates were used as control mice.

The care of the animals and all experimental procedures were conducted in accordance with the guidelines for animal experiments of Kyoto University and Nagoya City University and were approved by the Animal Research Committee of Kyoto University and Nagoya City University.

### 2.2 Diets

In all experiments, mice were fed with normal chow (NC) diet (3.4 kcal/g, fat 12 kcal%, CLEA Japan, Tokyo, Japan) until experiments. During experimental period, mice were fed either with NC, long-chain triglyceride (LCT) diet (protein 20 kcal%; carbohydrate 35 kcal%; fat 45 kcal%: Research Diets, New Brunswick, NJ; No. D12492), or MCT diet (protein 20 kcal%; carbohydrate 35 kcal%; fat 45 kcal%: Research Diets; No. D08041701), unless otherwise mentioned. MCT diet contains 60% of octanoate and 40% of decanoate. The nutritional composition of each experimental diet is listed in **Table 1**. The composition of the diet with different percentage of LCT/MCT is listed in the **Supplementary Table 1**. The fatty acid composition of NC is shown in the **Supplementary Table 2**.

**Table 1:** Nutrient composition of each diet.

Diet	NC diet	LCT diet	MCT diet
Nutrient composition			
Protein, %	29	20	20
Carbohydrate, %	59	35	35
Fat, %	12	45	45
Energy, kcal/g	3.44	4.73	4.73
Ingredients, g/100g (% kcal)			
Casein		23.3 (19.7)	23.3 (19.7)
L-cystine		0.3 (0.3)	0.3 (0.3)
Corn starch		8.5 (7.2)	8.5 (7.2)
Maltodextrine		11.7 (9.9)	11.7 (9.9)
Sucrose		20.2 (17)	20.2 (17)
Cellulose		5.8 (0)	5.8 (0)
Vitamin mix	0.9 (0)	1.4 (1.0)	1.4 (1.0)
Mineral mix	7.1 (0)	5.2 (0)	5.2 (0)
Soy bean oil		2.9 (5.6)	2.9 (5.6)
Lard		20.7 (39.4)	0 (0)
MCT oil		0 (0)	20.7 (39.4)

### 2.3 Food intake experiments

Mice were housed individually in cages throughout the experiment. Food intake was measured by weighing the food pellets. Small pellet fragments that fell onto the cage floor were collected as much as possible and included in the weight measurement. Energy intake was calculated by multiplying the weight of each food sample by its energy content per unit weight.

#### 2.3.1 Food intake in WT mice

After 10 weeks of NC diet feeding, mice were divided into three groups. One group was subjected to diet substitution from NC diet to LCT, and the other was to MCT diet. The remaining group continued on NC diet. After the switching of the diet, energy intake was measured for consecutive five days.

#### 2.3.2 Food intake in ghrelin-Tg mice

The diet of ghrelin-Tg mice was switched from NC to MCT diet at 10-week-old. After the switching of the diet, cumulative energy intake was measured every one week for four weeks.

#### 2.3.3 Continuous subcutaneous infusion of ghrelin to WT mice

Rat ghrelin (Peptide Institute, Osaka, Japan) solution of 0.18 µg/µl, 0.34 µg/µl, and 0.7 µg/µl were prepared for ghrelin infusion at rates of 0.9, 0.17, and 0.35 μg/hr, respectively and filled into a mini-osmotic pump (Alzet model 2001; Alza, Palo Alto, CA). The total filling volume of the mini-osmotic pump is 100 μl, and the infusion rate from the pump is 0.5 μl/hr. After 10 weeks of NC diet feeding, the mini-osmotic pump was implanted subcutaneously in the mid-scapular region of WT mouse. After the implantation, mice were divided into three groups according to diet (NC, LCT, or MCT, **Supplementary Figure 1**). The cumulative energy intake was determined by measuring the food intake of each mouse over five days and calculating the average for each group. The experiments were conducted with different cohorts for each of the NC, LCT, and MCT diets.

For the experiment using different lipid mix diets, WT mice were fed with five different lipid mix diets with decreasing amounts of MCT (40, 20, 10, 5, and 0%) substituted by increasing amount of LCT (0, 20, 30, 35, and 40%) (**Table 1**; **Supplementary Table 1**). Ghrelin was administered by the same paradigm as above-mentioned, but only at a dose of 0.35 μg/hr. Experiments were conducted on different group of mice for each different lipid mix diet.

#### 2.3.4 Acute intraperitoneal injection of ghrelin to WT mice

Based on the results of preliminary studies, the dose of ghrelin for this experiment was determined to be 360 μg/kg. After 10 weeks of NC diet feeding, mice were divided into three groups according to diet (NC, LCT, or MCT). After five days of diet switch, mice were injected intraperitoneally with saline or ghrelin after fasting for four hours. Energy intake was measured for 30 minutes following the injection.

#### 2.3.5 Intracerebroventricular infusion of NPY to WT mice

Based on the results of preliminary studies, the dose of neuropeptide Y(NPY) for this experiment was determined to be 100 pmol as a moderately effective dose and 500 pmol as a fully effective dose. NPY (Peptide Institute) at 100 pmol or 500 pmol was dissolved in artificial cerebrospinal fluid (aCSF) to a total volume of 1 μL, yielding solutions of 100 pmol/μL or 500 pmol/μL. WT mice were pre-implanted stereotaxically with cannulas into the right lateral ventricle under pentobarbital anesthesia before the diet switch. After five days of each diet feeding, mice were injected intracerebroventricularly either with aCSF or NPY at 100 or 500 pmol doses. Two-hour energy intake was measured following the injection.

### 2.4 Conditioned place preference test

For the measurement of food reward value, conditioned place preference (CPP) test was conducted. The CPP test was carried out in a three-chamber apparatus with a small middle chamber that connected two large side chambers (MED Associates, St. Albans, VT). Two large chambers differ in floor material and brightness, allowing the mice to distinguish between them by these cues and associate them with the placed food. CPP was performed as previously reported (18). Briefly, at experimental day 0, mice were allowed to move freely in the three chambers for 20 minutes. At days 1-3, mice were confined to one large chamber with NC diet for 30 minutes. Five hours later, they were confined to the other side chamber with NC, LCT, or MCT diet for 30 minutes. The mice recognized a single food pellet placed in center of the chamber during the 30 minutes conditioning period, as confirmed by the pellet being slightly crushed or wet with saliva. At day 4, mice were placed in the middle chamber and allowed to move freely between the three chambers for 20 minutes, similarly to that at day 0. CPP score was evaluated the difference in the time spent between the paired chambers, as follows: the time mice spent in the NC diet chamber subtracted from the time spent in the NC, LCT, or MCT diet chamber. The final CPP score is calculated by subtracting the CPP score of day 0 from the CPP score of day 4.

### 2.5 GH response to acute ghrelin administration

After 10 weeks of NC diet feeding, mice were divided into three groups according to diet (NC, LCT, or MCT). After five days of the diet switch, ghrelin was administered at a dose of 120 or 360 μg/kg by tail vein injection to mice. Blood was collected from retro-orbital vein 30 minutes after the injection and GH concentration was measured.

### 2.6 Measurement of plasma ghrelin, des-acyl ghrelin, leptin, and GH concentrations

Blood samples of mice for the measurement of plasma ghrelin or des-acyl ghrelin concentration were collected from retro-orbital venous on *ad-libitum* feeding in the morning. Samples were immediately transferred to chilled polypropylene tubes containing Na_2_EDTA (1mg/ml) and aprotinin (Ohkura pharmaceutical, Inc., Kyoto, Japan) and then centrifuged at 4°C. Separated plasma was immediately added with hydrogen chloride at the final concentration of 0.1 N. Ghrelin or des-acyl ghrelin was measured using a fluorescence enzyme immunoassay kit (Tosoh Corp. Tokyo, Japan). This assay kit is capable of distinguishing and measuring ghrelin and des-acyl ghrelin. The calibration curve range for ghrelin and des-acyl ghrelin in this assay system were 0.25–230 and 1.96-810  fmol/ml, respectively. The inter-assay coefficients of variation were 2.9 and 3.1% for ghrelin and des-acyl ghrelin, respectively (19). Plasma leptin and GH levels were measured by enzyme-linked immunosorbent assay kit (Morinaga Institute of biological science, Yokohama, Japan) and enzyme immuno-assay kit (SPI-BIO, Bonde, France), respectively.

### 2.7 Analysis of mRNA expression

The whole hypothalamus was quickly removed from decapitated mice two hours after ghrelin intraperitoneal injection. Removed hypothalamic tissues were snap frozen in liquid N_2_, stored at -80°C, and homogenized using BioMasherII (Nippi, Tokyo, Japan). The total RNA was extracted using the PureLink^®^ RNA Mini Kit (Thermo Fisher Scientific, Waltham, MA) with additional DNase treatment and was reverse-transcribed by High-Capacity cDNA Reverse Transcription Kit^®^ (Thermo Fisher Scientific) according to manufacturer’s instructions. The mRNA expression was measured by quantitative RT-PCR using probe and primer sets for each gene (Integrated DNA Technologies, Coralville, IA) and ABI 7500 Fast Real-Time PCR Systems (Thermo Fisher Scientific). Glyceraldehyde 3-phosphate dehydrogenase (GAPDH) gene was used as an internal control. The primer and probe sequence for each gene are shown in **Supplementary Table 3**.

### 2.8 Statistical analysis

Data are expressed as means ± SEM (± SD). Differences between two groups were assessed using unpaired two-tailed *t*-tests. For multiple group comparisons, data were analyzed with one-way analysis of variance (ANOVA) followed by *post-hoc* Bonferroni correction or non-paired *t*-test as appropriate. For experiments with repeated measurements within the same group, data were analyzed using repeated measures two-way ANOVA followed by *post-hoc* Bonferroni correction. *P* values less than 0.05 were considered to be statistically significant.

## 3 Results

### 3.1 Food intake and plasma concentrations of feeding-related hormones in WT mice fed each diet

**Figure 1:**
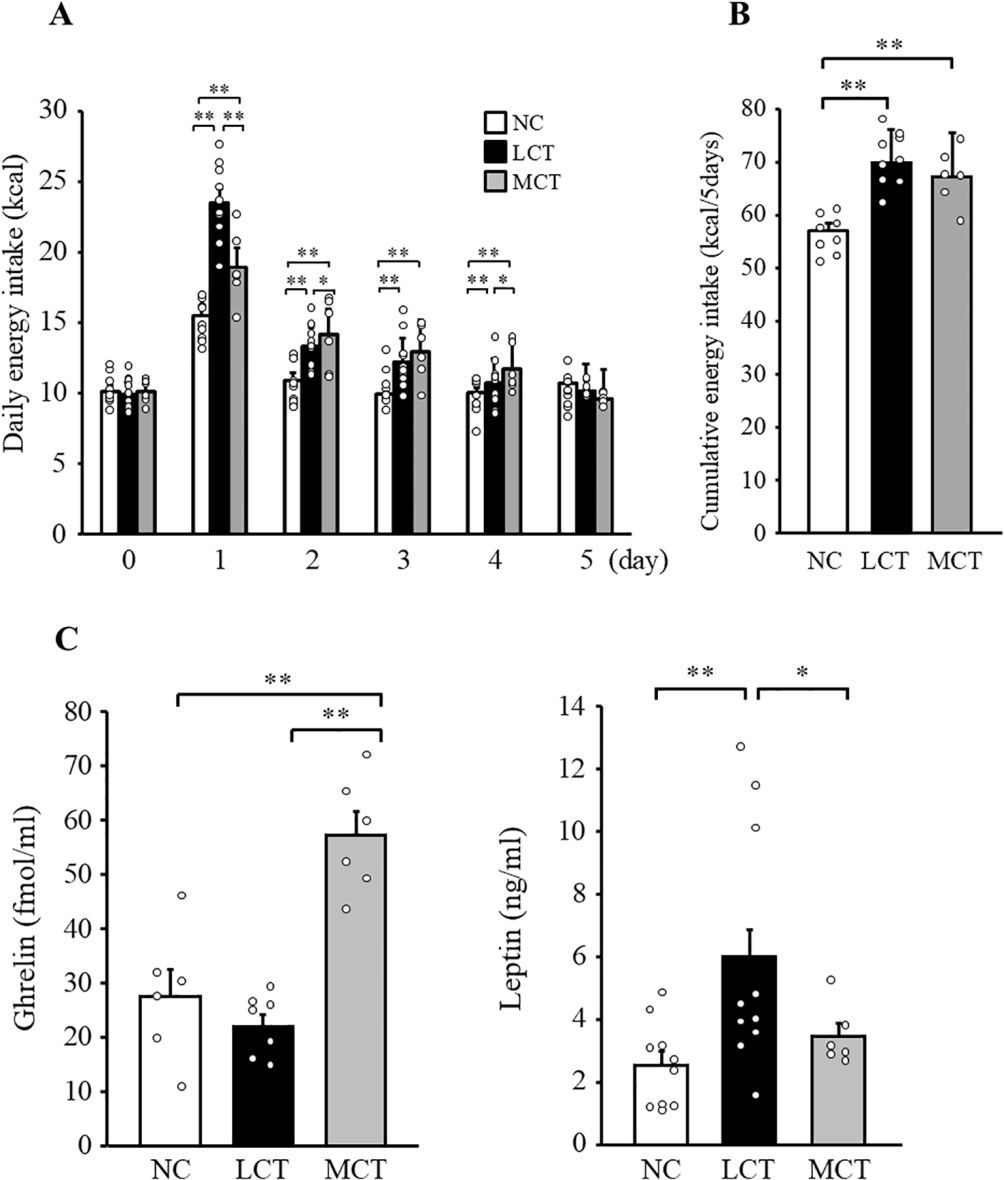
Effects of dietary MCT on energy intake and appetite regulating hormones in WT mice. Daily energy intake was measured from day 0 to day 5 **(A)** and cumulative energy intake from day 1 to day 5 were calculated **(B)**. Plasma ghrelin and leptin concentrations were measured in *ad-libitum* fed mice on day 5 **(C)**. n=8-11/group. Repeated measures two-way ANOVA followed by *post-hoc* Bonferroni correction were used for **(A)** and one-way ANOVA followed by Bonferroni multiple comparison tests were used for **(B, C)**. *p < 0.05, **p < 0.01.

We changed diet from NC to either LCT or MCT diet and assessed the daily energy intake in WT mice for five days. Energy intake of NC diet on day 0 was comparable between mouse groups allocated, but not started with LCT or MCT (**Figure 1A**, NC; 10.1 ± 0.4 (± 0.9) kcal, LCT; 9.9 ± 0.4 (± 1.1) kcal, MCT; 10.1 ± 0.3 (± 0.8) kcal, *p* = 0.84). Although energy intake on the first day, after the switch to high fat diet, was increased both in LCT and MCT-fed groups, mice given MCT ate significantly smaller amount than those fed with LCT (**Figure 1A**, NC; 15.5 ± 0.6 (± 1.2) kcal, LCT; 23.5 ± 0.9 (± 2.5) kcal, MCT; 18.9 ± 1.0 (± 2.3) kcal, LCT vs. MCT; *p* = 6.07×10^-27^, 95% confidence interval; -7.53 to -1.69, effect size; 1.75). No difference was observed during the other four days from day 2 through day 5. Cumulative energy intake during the five days period after switching of diet was also comparable between LCT- and MCT-fed mice (**Figure 1B, NC**; 57.0 ± 1.4 (± 3.5) kcal, LCT; 69.9 ± 1.7 (± 5.5) kcal, MCT; 67.2 ± 2.2 (± 5.3) kcal, NC vs. LCT; *p* = 5.5×10^-6,^ NC vs. MCT; *p* = 1.2×10^-4^). The body weight of WT mice fed LCT and MCT for five days were 24.9 ± 0.4 (± 1.2) g and 23.7 ± 0.4 (± 1.3) g, respectively, while that fed NC continuously was 23.7 ± 0.4 (± 1.1) g. MCT-fed mice exhibited more than 2.5-fold higher plasma ghrelin concentration compared with LCT-fed mice (**Figure 1C**). On the other hand, plasma leptin concentration measured on day 5 was significantly lower in MCT-fed mice compared with LCT-fed mice. Since ghrelin and leptin are orexigenic and anorexigenic hormones, respectively, these results suggest a possibility of impaired ghrelin signaling, leptin hypersensitivity, or both.

### 3.2 Food intake and body weight of ghrelin-Tg mice fed NC or MCT diet

**Figure 2:**
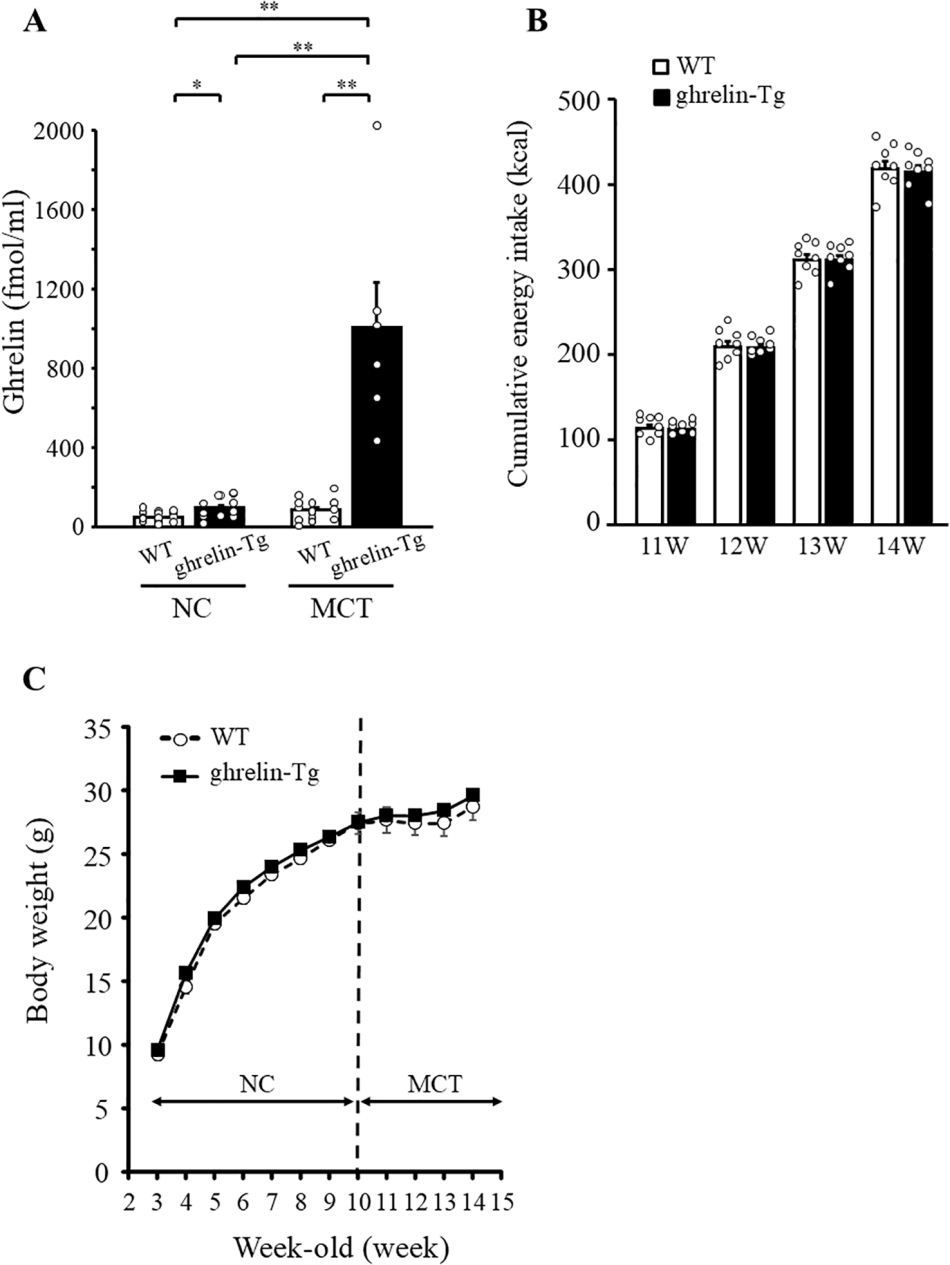
Effects of dietary MCT on energy intake and body weight in ghrelin transgenic (ghrelin-Tg) mice. Plasma ghrelin concentration was measured in ghrelin-Tg or WT mice fed with both NC diet (at 10-week-old) and MCT diet for one week (at 11-week-old) **(A)**. Cumulative energy intake from 11- to 14-weeks were measured **(B)**. Body weights were measured weekly under NC and MCT diet conditions **(C)**. n=8/group. One-way ANOVA followed by Bonferroni multiple comparison tests were used for **(A)** and repeated measures two-way ANOVA followed by *post-hoc* Bonferroni correction were used for **(B, C)** *p < 0.05, **p < 0.01.

There was a possibility that the increase in blood ghrelin concentration caused by MCT intake was not high enough to promote food intake in **Figure 1C**. To investigate this possibility, we conducted experiments using ghrelin-Tg mice with even higher blood ghrelin concentrations. Under the NC diet, ghrelin-Tg mice showed about two-fold increase in plasma ghrelin concentration compared with WT mice (**Figure 2A**, WT; 48.2 ± 5.9 (± 24.8) fmol/ml, ghrelin-Tg; 93.5 ± 15.2 (± 50.3) fmol/ml, *p* = 0.048). Plasma des-acyl ghrelin concentration in ghrelin-Tg mice was remarkably increased to more than 170-fold higher than that seen in WT mice on NC diet (**Supplementary Figure 2**, WT; 159.8 ± 16.1(± 70.2) fmol/ml, ghrelin-Tg; 28319.8 ± 1328.5 (± 4602) fmol/ml, *p* = 4.48×10^-22^). One week after switch from NC to MCT diet at 10th week, MCT feeding tended to increase plasma ghrelin levels in WT mice (**Figure 2A**). Plasma ghrelin concentration in MCT-fed ghrelin-Tg mice was remarkably increased to more than 10-fold higher than that seen in ghrelin-Tg mice on NC diet (**Figure 2A**, NC; 93.5 ± 15.2 (± 50.3) fmol/ml, MCT; 1005.3 ± 225.8 (± 505.0) fmol/ml, *p* =1.9×10^-3^). Nevertheless, weekly cumulative energy intake after switch from NC to MCT at 10th week did not differ between the genotypes (**Figure 2B**). The body weight of ghrelin-Tg mice was 9.5 ± 0.4 (± 1.8) g at 3-week-old when the experiment started, which was comparable to that of WT at 9.2 ± 0.4 (± 2.1) g. Body weight curves were indistinguishable and there was no difference in body weight between WT and ghrelin-Tg both before and after the switch from NC to MCT (**Figure 2C**).

### 3.3 Food intake during continuous ghrelin administration in WT mice fed each diet

**Figure 3:**
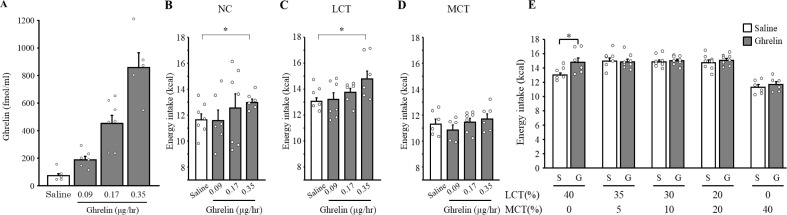
Effects of MCT diet on orexigenic effect of continuous ghrelin administration. Plasma ghrelin concentration after continuous administration of ghrelin at each dose for five days were measured in mice fed with NC diet **(A)**. Five days energy intake during the ghrelin administration at each dose in each diet **(B-D)**. Mice were fed with five different lipid mix diets with decreasing amounts of MCT (40, 20, 10, 5, and 0%) substituted by increasing amount of LCT (0, 20, 30, 35, and 40%). Five days energy intake during the ghrelin administration at a dose of 0.35 μg/hr in each diet **(E)**. n=5-8/group. One-way ANOVA followed by Bonferroni multiple comparison tests were used for **(B–D)**, and non-paired two-tailed *t*-tests were undertaken for **(E)** *p < 0.05 vs. saline.

To address the effect of continuously administered ghrelin on food intake, we treated mice with ghrelin by mini-osmotic pumps and measured energy intake. When WT mice on the NC diet were continuously infused with ghrelin at doses of 0.09, 0.17, or 0.35 µg/hr subcutaneously for five days (**Supplementary Figure 1**), the plasma ghrelin concentration was increased in a dose-dependent manner (**Figure 3A**). In mice administered with ghrelin at the highest dose of 0.35 µg/hr, plasma ghrelin concentration was increased up to 858.1 ± 105.8 (± 236.5) fmol/ml, tantamount to the level reached by the ghrelin-Tg mice fed with MCT diet. We then implanted ghrelin-infusing pumps subcutaneously in WT mice and initiated either LCT or MCT diet on the same day (**Supplementary Figure 1**). Infusion of ghrelin significantly stimulated energy intake both in mice fed with the NC (**Figure 3B**, p = 0.036 vs. saline) and the LCT diets (**Figure 3C**, p =0.021 vs. saline). In contrast, however, no significant change in food intake was observed in mice fed MCT following ghrelin administration (**Figure 3D**, p = 0.51 vs. saline).

To examine the minimal concentration of MCT contained in the diets at which ghrelin-induced hyperphagia ceases to be observed, we prepared five different types of 40%kcal fat diet with varying MCT percentages, ranging from the lowest 0%kcal up to the highest 40%kcal of the total energy (**Supplementary Table 1**). Ghrelin was continuously administered at a dose of 0.35 μg/hr and energy intake was measured for five days. Ghrelin significantly increased energy intake in mice fed with LCT diet (**Figure 3E**, p = 0.021 vs. saline). Notably, the orexigenic effect of ghrelin was not observed even by the presence of as low as 5%kcal containment of MCT within the total 40%kcal fat (**Figure 3E**, p = 0.90 vs. saline). This attenuation was observed in 10%-, 20%-, and 40%-MCT containing diets (**Figure 3E**, p = 0.72, *p* = 0.49, *p* = 0.51 vs. saline, respectively).

### 3.4 Reward value of each diet

**Figure 4:**
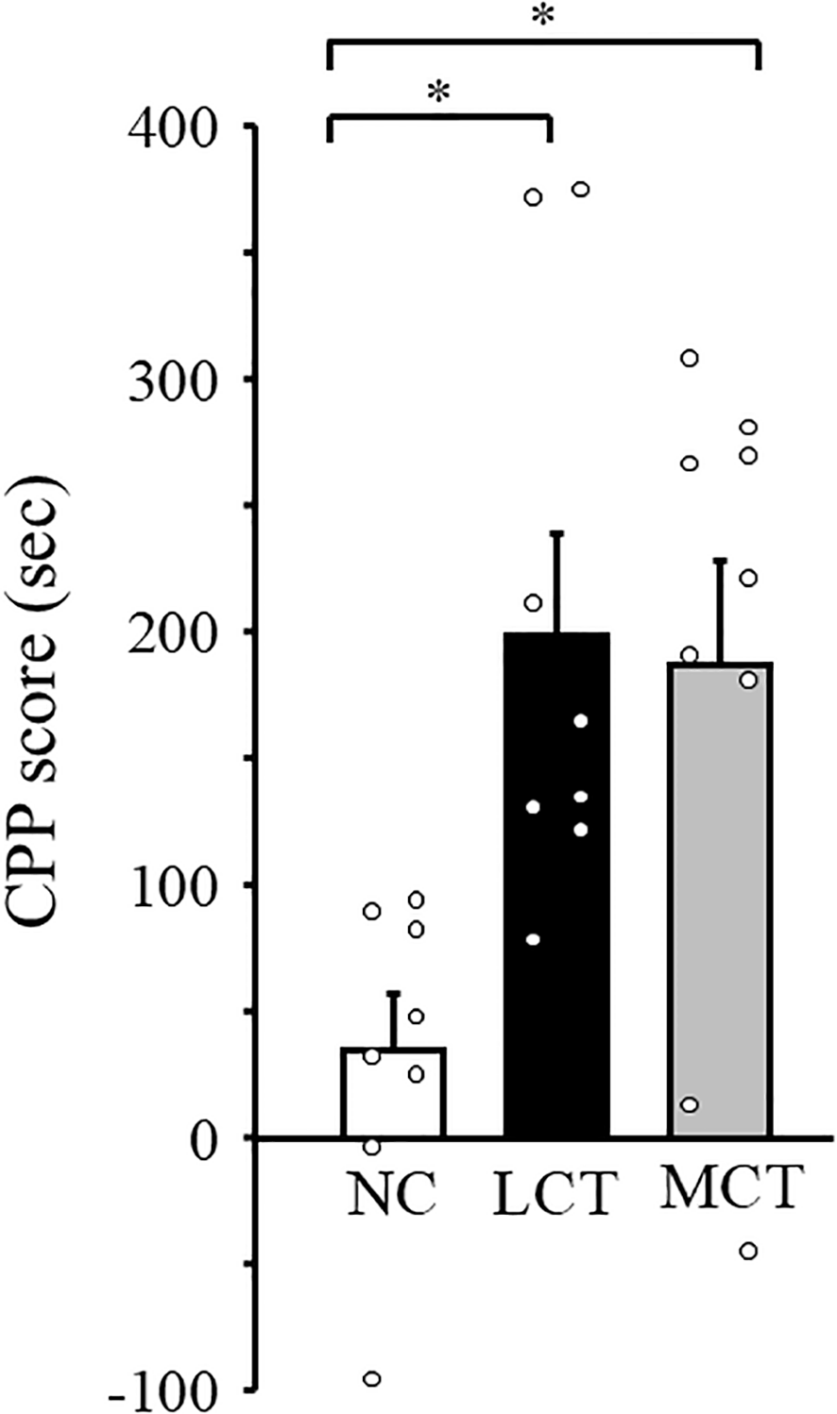
Measurement of food reward value of MCT diet in WT mice. CPP score for NC, LCT, and MCT diet were measured by CPP test. The CPP score in the Y-axis is calculated by subtracting the CPP score of day 0 from the CPP score of day 4 for each diet. n=8-9/group. *p < 0.05 by one-way ANOVA followed by Bonferroni multiple comparison tests.

The fact that no orexigenic effect of ghrelin was observed when feeding a diet containing only 5%kcal from MCT has raised a possibility of the avoidance behavior of mice to MCT diet. To exclude the possibility of taste aversion, we evaluated the reward value of the MCT diet using CPP test. CPP score for MCT diet was significantly higher compared with that for NC diet, and was comparable with that for LCT diet (**Figure 4**). These results indicate that MCT diet is as palatable as LCT diet.

### 3.5 Gene expression of appetite-regulating neuropeptide in the hypothalamus following ghrelin administration in WT mice fed each diet

**Figure 5:**
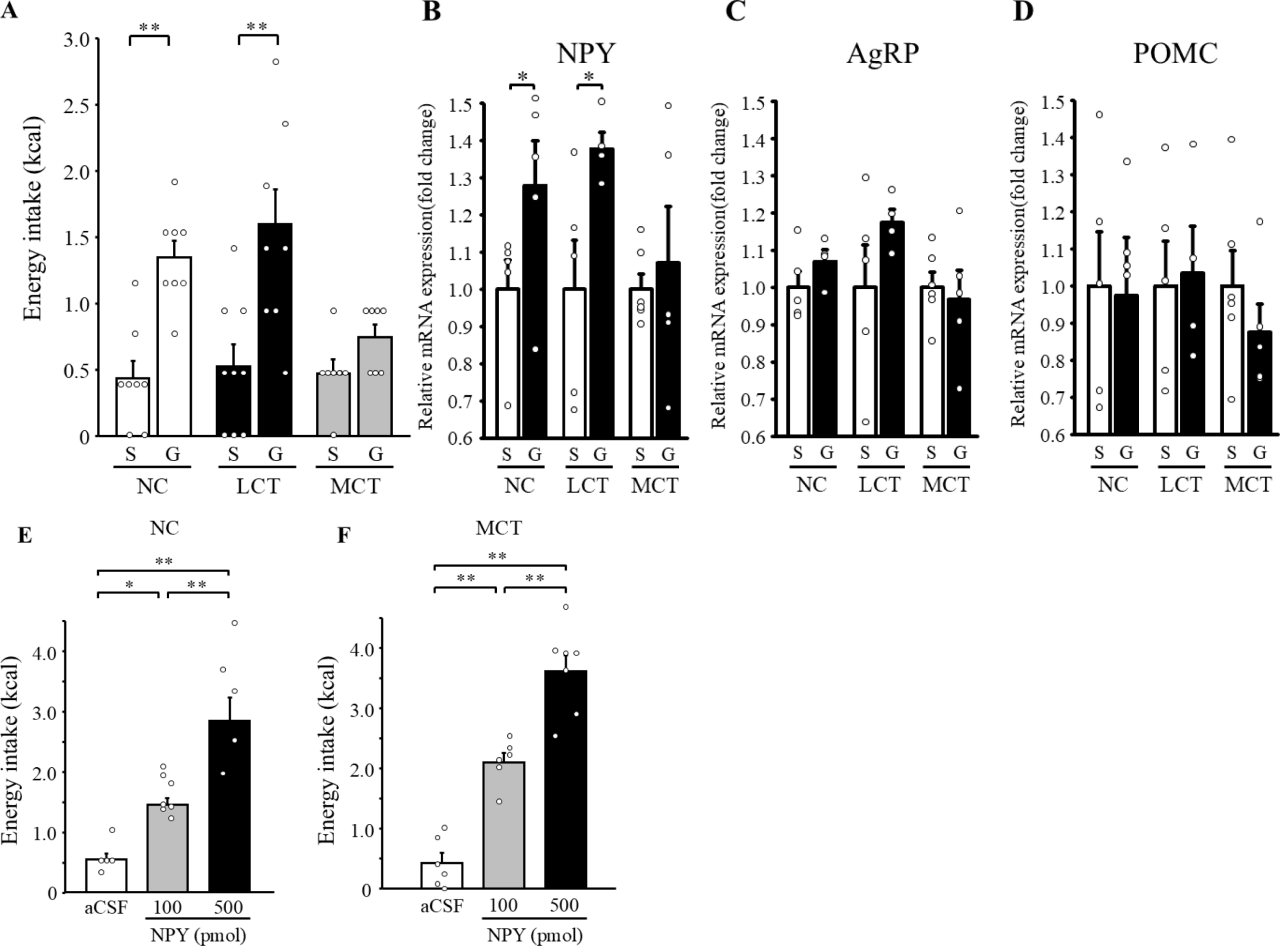
Analysis of the role of central NPY in attenuated ghrelin signaling by the MCT diet. Mice were injected intraperitoneally either with saline (S) or ghrelin (G, 360 µg/kg) after four-hour fasting. Thirty minutes energy intake following the injection **(A)**. Neuropeptide (NPY, AgRP, and POMC) gene expression in the hypothalamus was analyzed. Gene expression is shown in the relative to saline-treated mice **(B-D)**. After five days of each diet feeding, mice were injected intracerebroventricularly either with aCSF or NPY at 100 or 500 pmol doses. Two-hour energy intake was measured following the injection **(E, F)**. n=7-9/group **(A)**, or n=5-7/group **(B-F)**. Non-paired *t*-tests were used for **(A–D)**, and one-way ANOVA followed by Bonferroni multiple comparison test was undertaken for **(E, F)** *p < 0.05, **p < 0.01.

In mice fed either with NC, LCT, or MCT diet for five days, ghrelin was injected intraperitoneally at a dose of 360 µg/kg. Similar to chronic administration, acute ghrelin injection significantly stimulated energy intake during the following 30 minutes under the NC diet (**Figure 5A**, p =8.1×10–^4^ vs. saline). This increase in energy intake by ghrelin was also conserved in mice fed with the LCT diets (**Figure 5A**, p =8.3×10^-3^ vs. saline). The orexigenic effect of ghrelin was, however, again not observed in mice fed with the MCT diet (**Figure 5A**, p = 0.073 vs. saline).

We examined the mRNA expression of appetite-regulating neuropeptides in the hypothalamus of mice 120 minutes after ghrelin injection. The expression of NPY, an important downstream mediator of ghrelin-induced increase in food intake, was significantly increased by ghrelin both in NC- and LCT-fed mice (**Figure 5B**, NC; *p* = 0.048 vs. saline, LCT; *p* = 0.045 vs. saline), but this was not the case with the MCT diet (**Figure 5B**, p = 0.63 vs. saline). The hypothalamic mRNA expression of agouti-related protein (AgRP) and pro-opiomelanocortin (POMC), which is orexigenic and anorexigenic neuropeptide, respectively, did not differ among the groups (**Figures 5C, D**, AgRP: NC; *p* = 0.13 vs. saline, LCT; *p* = 0.12 vs. saline, MCT; *p* = 0.65 vs. saline, POMC: NC; *p* = 0.91 vs. saline, LCT; *p* = 0.85 vs. saline, MCT; *p* = 0.35 vs. saline).

### 3.6 Food intake following intracerebroventricular administration of NPY in WT mice

To gain further insight into the mechanism underlying MCT-induced suppression of the ghrelin’s orexigenic effect, we examined the effect of NPY administered intracerebroventricularly. As reported, centrally administered NPY at doses of 100 and 500 pmol significantly increased energy intake in mice fed with the NC diet (**Figure 5E**, aCSF vs. NPY100; *p* = 0.028, aCSF vs. NPY500; *p* = 1.6×10^-5^, NPY100 vs. NPY500; *p* = 1.2×10^-3^). Of note, significant increase of energy intake by NPY injection was similarly observed even under the MCT diet (**Figure 5F**, aCSF vs. NPY100; *p* = 1.6×10^-4^, aCSF vs. NPY500; *p* = 2.9×10^-8^, NPY100 vs. NPY500; *p* = 3.1×10^-4^). Contrary to attenuated response to ghrelin, the activity of the central NPY receptor signaling pathway was preserved even in mice fed with the MCT diet.

### 3.7 GH secretion following ghrelin administration in WT mice fed each diet

**Figure 6:**
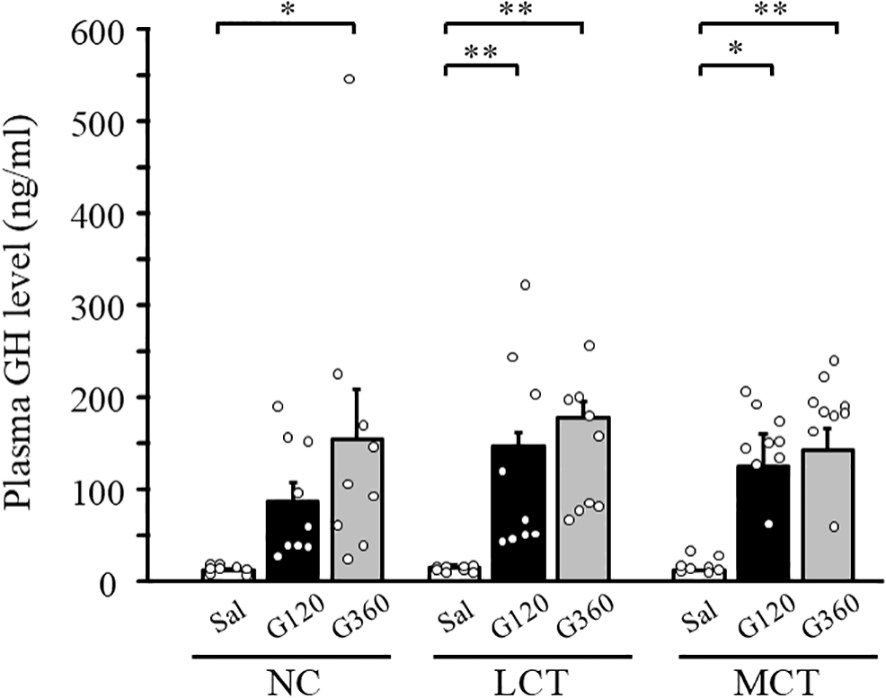
Effects of MCT diet on ghrelin-stimulated GH secretion. After five days of the diet switch, mice were injected intravenously either with saline (S) or ghrelin (G) at a dose of 120 or 360 μg/kg. Blood was collected 30 minutes following the injection and GH concentration was measured. n=8-9/group. *p < 0.05, **p < 0.01 by one-way ANOVA followed by Bonferroni multiple comparison tests.

It is well known that peripheral administration of ghrelin not only increases food intake but also promotes GH secretion. Therefore, we examined the effect of dietary MCT on ghrelin action with respect to GH secretion. Plasma GH levels were measured 30 minutes after the intravenous ghrelin injection in mice fed with each diet for five days. The plasma GH levels were significantly increased by ghrelin in mice fed with the NC or the LCT diet (**Figure 6**). Importantly, significant increase of GH levels by ghrelin was still preserved under the MCT diet (**Figure 6**). GH levels achieved by ghrelin administration were comparable among the diets (**Figure 6**).

## 4 Discussion

In the present study, we report that the orexigenic effect of ghrelin was not observed in mice fed a MCT diet. Although MCT feeding augments ghrelin production, increased ghrelin levels in WT or ghrelin-Tg mice is not associated with hyperphagia. Consistently, single dose injection or chronic infusion of ghrelin, which potently increases energy intake under NC or LCT diets, does not exert any effect under MCT diet. By a CPP test, we confirmed that the MCT diet, we used, is as palatable as the LCT diet with higher reward value compared with the NC diet, arguing against the secondary effect of taste aversion. Ghrelin-induced augmentation of hypothalamic NPY gene expression is not observed under MCT feeding, while central NPY injection leads to hyperphagia even under MCT diet, suggesting that ghrelin signaling upstream of NPY is attributable to the blunting ghrelin’s effect. Contrary to the orexigenic effect of ghrelin, its effect on plasma GH levels is not attenuated by MCT, indicating distinct pathways.

The notion has become widespread recently that nutrients affect hormonal secretion or sensitivity (20–22). Ghrelin secretion, for example, is determined by MCT diets which contains octanoate (10, 12). In the case of leptin, on the other hand, its anorexigenic and body weight-reducing effect are abolished by LCT diets, widely known as “leptin resistance” (23–25). In this context, whether orexigenic effect of ghrelin is influenced by dietary nutrients has never been addressed.

In the current study, plasma ghrelin level was increased, as expected, by 2.5-fold on the 5th day of MCT feeding. However, enhanced ghrelin level was not associated with increased energy intake, pointing to the potential development of impaired ghrelin signaling under the MCT diet. More importantly, when ghrelin was injected or continuously infused, enhancement of energy intake observed under NC or LCT diets did not occur under the MCT diet. These data suggest that the hormonal effects of ghrelin may be impaired during MCT intake.

Ghrelin, once diffused across the blood-brain barrier, binds to GHSR of the NPY neurons in the arcuate nucleus of the hypothalamus and exerts its orexigenic and body weight increasing effects (26–28). In line with this, centrally-administered GHSR antagonist blocks the effect of peripherally-administered ghrelin (29). Furthermore, central NPY-Y1 receptor antagonism attenuates ghrelin-induced hyperphagia, suggesting the importance of hypothalamic NPY-Y1 receptor pathway downstream of ghrelin (30).

In the current study, we clearly show that the NPY gene expression in the hypothalamus is increased by ghrelin injection under NC or LCT diet. Strikingly, augmentation of NPY expression is not observed when mice are fed with the MCT diet in parallel with an attenuation of ghrelin-induced hyperphagia. These data suggest the possibility that defective NPY induction caused by MCT is responsible for the failure to observe the orexigenic effect of ghrelin. Importantly, administration of NPY directly into the central nervous system stimulates energy intake even under MCT diet, demonstrating that the site of impaired ghrelin signaling resides at the level of NPY induction or upstream.

One possible explanation for impaired ghrelin signaling by MCT may be the secondary outcome of the changes of other hormones. For example, MCT reportedly raises the levels of anorexigenic hormones, such as peptide YY and leptin in humans (31). In fact, leptin, potentially through its specific suppression of ghrelin-induced Ca^2+^ flux in NPY neurons (32), serves as a counter-regulatory hormone to ghrelin (3, 32). Since plasma leptin concentration in MCT-fed mice is lower than that in LCT-fed mice in the present study, leptin itself may not be involved in the defective NPY induction by ghrelin in our experimental settings. There is also a report demonstrating that MCT intake, particularly decanoic acid, promotes GLP-1 secretion in mice (33). The increased GLP-1 secretion from the intestine due to dietary MCT may have antagonized or attenuated ghrelin’s orexigenic effects. Furthermore, other unknown hormones increased by the MCT feeding might possibly be involved.

Nutrient availability may influence GHSR regulation in the central nervous system. Indeed, previous reports have shown that feeding mice LCT diet for 12 weeks reduces GHSR gene expression in the hypothalamus, potentially contributing to diminished ghrelin effects (34, 35). To our knowledge, there are no reports on GHSR regulation under MCT feeding, but it cannot be excluded that dietary MCT may have reduced GHSR expression in the hypothalamus. MCT intake may have also reduced ghrelin stability or bioavailability. These mechanisms remain possible, even though the GH-secreting effect was maintained under the MCT diet, because the GH-secreting effect and the appetite-promoting effect are considered to be distinct.

Liver-expressed antimicrobial peptide (LEAP)-2 is an endogenous GHSR antagonist or inverse agonist (36, 37). Systemic or central administration of LEAP-2 to WT mice suppressed the feeding promoting effect of ghrelin administration (36–38). Blood LEAP-2 concentrations have been reported to fluctuate in relation to obesity levels and obesity-related parameters (39). However, the blood LEAP-2 concentration during MCT intake remains unknown and requires further investigation.

We show that GH release in response to ghrelin is maintained even under the MCT diet. Ghrelin acts on the GH-releasing hormone (GHRH) neurons to induce GH secretion (40). Although both are located in the same arcuate nucleus of the hypothalamus, GHRH neurons and NPY neurons constitute a distinct subset of neuronal cells (41). Since ghrelin-GHRH-GH axis is also functionally distinct from NPY signaling (42), MCT-induced impaired ghrelin signaling can be viewed as both target cell-specific and physiologic function-specific.

MCT diet in the present research is composed of 60% octanoate and 40% decanoate. Ingestion of glyceryl tridecanoate increases the production of n-decanoyl ghrelin (C10:0-ghrelin) in the stomach of mice (12). Decanoyl ghrelin is reported to have similar potency to ghrelin, which is endogenously mainly octanoylated, upon application to GHSR-expressing cells and to display with nearly identical dose-response relationships to ghrelin *in vitro* (43). It remains unclear which of the two MCFA-esterified ghrelin is predominant in the blood of our experimental animal and which of the two is more predominantly responsible for the impaired ghrelin signaling. Warren et al. reported that decanoate inhibits mammalian target of rapamycin complex 1 (mTORC1) signaling pathway (44). Based on this, decanoate may be involved in the inhibition of ghrelin effect to some extent, because ghrelin-induced hyperphagia are controlled by a central nervous system via mTORC1 pathway (45).

We show that ghrelin-induced hyperphagia was no longer observed with only 5%kcal addition of MCT out of the total of 40%kcal fat in LCT predominant high fat diet. This better supports the hypothesis that a trace amount of MCT-derived mediator specifically interferes with the ghrelin signaling pathway, rather than MCT and LCT-derived lipids compete with each other on metabolic flux as sources of energy.

There are reports showing that in diet-induced obese mice, ghrelin-stimulated enhancement of food intake is attenuated (34, 46–48), leading to the concept or “ghrelin resistance” in obesity. The “ghrelin resistance” in those studies are thought to be the result of the metabolic consequence of body weight gain after long-time exposure to LCT diet, rather than by the consumption of LCT diet per se (34). In fact, in the present study, we did not observe impaired ghrelin signaling in mice fed with the LCT diet for five days.

What we have observed here, on the other hand, is that just a short term MCT diet abolished ghrelin-induced hyperphagia, which we call “impaired ghrelin signaling” here. Based on stark differences of the length of the diet, and the presence or absence of obesity, impaired ghrelin signaling by MCT we report here is totally distinct from what have been reported in LCT-induced obese animal models (49, 50). The effects of long-term MCT ingestion on food intake remain unknown, which is an important consideration for future clinical application.

In the present research, we could not find increased energy intake or body weight in the ghrelin-Tg mice despite marked increase of plasma ghrelin concentration. To elucidate the role of ghrelin in the regulation of food intake, we have been trying to generate variable models of transgenic mice overexpressing ghrelin using different promotors. These mice did not show any phenotype in growth or food intake, since almost all of these animals produced only des-acyl ghrelin rather than ghrelin (17, 51). After the identification of GOAT in 2008, we have succeeded in generating ghrelin-Tg mice overexpressing both mouse GOAT and des-acyl ghrelin in the liver, as used in the present study. Although the plasma ghrelin concentration of ghrelin-Tg mice was increased approximately two-fold compared with that of WT mice under the NC diet, there were no difference in phenotype between the two genotypes. There are some reasons why the ghrelin-Tg mice showed no apparent phenotypic changes in body weight and food intake under the NC diet. Firstly, the ghrelin-Tg mice had been constitutively exposed to high level of ghrelin from their birth, which may result in an attenuation of the ghrelin effect. Secondly, a markedly increased plasma des-acyl ghrelin in NC-fed ghrelin-Tg mice may inhibit the orexigenic effect of ghrelin, since there is a report showing potential inhibitory effect of des-acyl ghrelin on the action of peripherally-injected ghrelin in rats (52).

The limitations of this study include the following. First, we examined the difference in food intake by comparing MCT feeding with LCT feeding. The LCT diet and MCT diet used in the experiment share the same ingredient composition and material sources, except for the difference between LCT and MCT. However, ideally, mice should be fed completely the same diet when comparing food consumption. Second, we conducted a series of experiments only in male mice. Therefore, it remains unclear whether dietary MCT similarly attenuates the feeding effect of ghrelin in female mice. Third, we examined gene expression analysis using whole hypothalamus tissue and found no difference in POMC and AgRP gene expression across the groups. This approach may dilute the signal from the arcuate nucleus, a specific site responsible for appetite-promoting effects of ghrelin. Forth, since we collected experimental data with food placed on top of the mouse cages, we were not completely blinded to the group assignments. We cannot exclude the possibility that this introduced potential bias into the experimental results. Furthermore, the results of this study are purely exploratory due to the insufficient sample size.

Through a number of basic and clinical studies, it is established that MCT ingestion reduces body fat and body weight (53–55). The postulated mechanism has been that MCFA derived from MCT is more prone to oxidation rather than tissue storage. These data have provided supporting evidence for the beneficial metabolic effects of dietary MCT. In the present study, we clearly show a novel antagonism of the ghrelin signaling by MCT. Our data heretofore provide an unraveled mechanism through which MCT diet protects against weight gain. We also show that MCT does not interfere with ghrelin’s GH-secreting effect. Since GH is a hormone that acts positively to maintain or increase skeletal muscle mass, MCT diet could serve as an ideal modulator that controls body weight and composition in a favorable manner.

Several studies have suggested that obesity is associated with decreased circulating ghrelin level, and impaired ghrelin-induced food consumption (56–58). Conversely, weight loss leads to a restoration of plasma ghrelin concentration and its sensitivity (59). This might possibly contribute to the exaggerated body weight increase following a period of dietary restriction. In fact, weight regain after calorie restriction is reduced in ghrelin-deficient mice (59). In addition, inhibition of GOAT or GHSR, or down regulation of ghrelin signaling by ghrelin vaccination reduce body weight in mice (60–62). However, strategy to inhibit ghrelin signaling may have shortcomings; increased stress or anxiety, decreased learning, memory or motivation (63–66). Future studies are awaited to prove that MCT or its derivative may be a safe yet effective measures to treat obesity disease.

This study reveals a novel interaction between dietary nutrient and hormone signaling by showing the effect of ghrelin on food intake under MCT feeding. These data provide a mechanistic basis that enables the future development of MCT-related foods or drugs for the treatment of obesity and the metabolic syndrome.

## Data Availability

The datasets presented in this study can be found in online repositories. The names of the repository/repositories and accession number(s) can be found in the article/**Supplementary Material**.

## Supplementary material

Supplementary Figure 1 The method for the continuous ghrelin administration. Mini-osmotic pump was implanted in the dorsal subcutaneous region and ghrelin was continuously administered for five days at doses of 0.09, 0.17, or 0.35 μg/hr. On the same day of the pump implantation, the diet was either continued on NC diet or substituted with LCT or MCT diet. n=5-7/group.

Supplementary Figure 2 Plasma des-acyl ghrelin concentration in ghrelin-Tg and WT mice. Plasma ghrelin concentration was measured in ghrelin-Tg and WT mice fed with NC diet. The inserted panel is magnified view of bar graph of WT mice. n=8/group. ***p* < 0.01 by non-paired *t*-tests.
