# Supplementary material for: Dietary medium chain triglycerides impairs orexigenic action of ghrelin in mice
Source: Front Endocrinol (Lausanne). 2026 Jan 5;16:1690761. doi: 10.3389/fendo.2025.1690761 (PMC12812557; doi:10.3389/fendo.2025.1690761)
Supplement: Supplementary file 1 [file Table1.docx]

|  | | LCT 35/MCT 5 | | LCT 30/MCT 10 | | | LCT 20/MCT 20 | | |
| --- | --- | --- | --- | --- | --- | --- | --- | --- | --- |
| Nutrient composition | |  | |  | | |  | | |
|  | Protein, % | 20 | | 20 | | | 20 | | |
|  | Carbohydrate, % | 35 | | 35 | | | 35 | | |
|  | Fat, % | 45 | | 45 | | | 45 | | |
|  | Energy, kcal/g | 4.73 | | 4.73 | | | 4.73 | | |
| Ingredients, g/100g (% kcal) | |  |  | |  |  | |  |  |
|  | Casein | 23.3 | (19.7) | | 23.3 | (19.7) | | 23.3 | (19.7) |
|  | L-cystine | 0.3 | (0.3) | | 0.3 | (0.3) | | 0.3 | (0.3) |
|  | Corn starch | 8.5 | (7.2) | | 8.5 | (7.2) | | 8.5 | (7.2) |
|  | Maltodextrine | 11.7 | (9.9) | | 11.7 | (9.9) | | 11.7 | (9.9) |
|  | Sucrose | 20.2 | (17.0) | | 20.2 | (17.0) | | 20.2 | (17.0) |
|  | Cellulose | 5.8 | (0) | | 5.8 | (0) | | 5.8 | (0) |
|  | Vitamin mix | 1.4 | (1.0) | | 1.4 | (1.0) | | 1.4 | (1.0) |
|  | Mineral mix | 5.2 | (0) | | 5.2 | (0) | | 5.2 | (0) |
|  | Soy bean oil | 2.9 | (5.6) | | 2.9 | (5.6) | | 2.9 | (5.6) |
|  | Lard | 18.1 | (34.5) | | 15.5 | (29.6) | | 10.3 | (19.7) |
|  | MCT oil | 2.6 | (4.9) | | 5.2 | (9.8) | | 10.4 | (19.7) |

Supplementary Table 1

Nutrient composition of the diet with different percentage of LCT/MCT
