# Supplementary material for: Dietary medium chain triglycerides impairs orexigenic action of ghrelin in mice
Source: Front Endocrinol (Lausanne). 2026 Jan 5;16:1690761. doi: 10.3389/fendo.2025.1690761 (PMC12812557; doi:10.3389/fendo.2025.1690761)
Supplement: Supplementary file 2 [file Table2.docx]

Supplementary Table 2

Fatty acid composition of NC diet (% of total fatty acid)

| Myristic acid (%) | 0.82 |
| --- | --- |
| Palmitic acid (%) | 16.25 |
| Palmitoleic acid (%) | 1.34 |
| Heptadecanoic acid (%) | 0.52 |
| Stearic acid (%) | 2.13 |
| Oleic acid (%) | 20.95 |
| Vaccenic acid (%) | 2.11 |
| Linoleic acid (%) | 44.44 |
| Alpha-linolenic acid (%) | 3.26 |
| Arachidic acid (%) | 0.49 |
| Eicosenoic acid (%) | 0.72 |
| Eicosapentaenoic acid (%) | 2.27 |
| Behenic acid (%) | 0.25 |
| Docosahexaenoic acid (%) | 1.29 |
| Lignoceric acid (%) | 0.18 |
| Tetraconsenoic acid (%) | 0.20 |
| Unidentified (%) | 2.78 |
