# Supplementary material for: Dietary medium chain triglycerides impairs orexigenic action of ghrelin in mice
Source: Front Endocrinol (Lausanne). 2026 Jan 5;16:1690761. doi: 10.3389/fendo.2025.1690761 (PMC12812557; doi:10.3389/fendo.2025.1690761)
Supplement: Supplementary file 3 [file Table3.docx]

Supplementary Table 3

Primer and probe sequence for real-time PCR analysis for NPY, AgRP, POMC, and GAPDH mRNAs

| NPY | |
| --- | --- |
|  | Forward: 5’-ACATCAATCTCATCACCAGACAG-3’ |
|  | Reverse: 5’-ACAAGTTTCATTTCCCATCACC-3’ |
|  | Probe: 5’-/56-FAM/CCCAGAACA/ZEN/AGGCTTGAAGACCCT/3IABkFQ/-3’ |
|  |  |
| AgRP | |
|  | Forward: 5’-GCTACAGGAAGCAGTCACG-3’ |
|  | Reverse: 5’-CAGAACACAACTCAGCAACA-3’ |
|  | Probe: 5’-/56-FAM/CAGGGCACA/ZEN/AGAGACCAGGACATC/3IABkFQ/-3’ |
|  |  |
| POMC | |
|  | Forward: 5’-TGAACATCTTTGTCCCCAGAG-3’ |
|  | Reverse: 5’-CTGAGCGACTGTAGCAGAATC-3’ |
|  | Probe: 5’-/56-FAM/AGAGACTAG/ZEN/GCCTGACACGTGGA/3IABkFQ/-3’ |
|  |  |
| GAPDH | |
|  | Forward: 5’-AATGGTGAAGGTCGGTGTG-3’ |
|  | Reverse: 5’-GTGGAGTCATACTGGAACATGTAG-3’ |
|  | Probe: 5’-/5HEX/TGCAAATGG/ZEN/CAGCCCTGGTG/3IABkFQ/-3’ |
