# Supplementary figures and images for: Dietary medium chain triglycerides impairs orexigenic action of ghrelin in mice
Source: Front Endocrinol (Lausanne). 2026 Jan 5;16:1690761. doi: 10.3389/fendo.2025.1690761 (PMC12812557; doi:10.3389/fendo.2025.1690761)

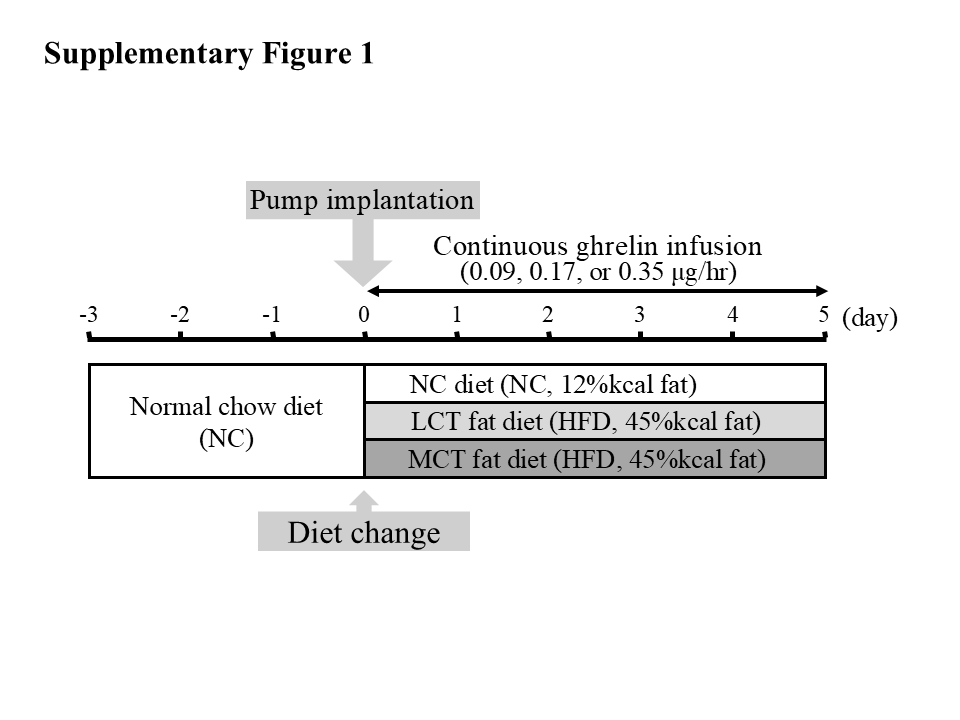

Supplement: Supplementary Figure 1 — The method for the continuous ghrelin administration. Mini-osmotic pump was implanted in the dorsal subcutaneous region and ghrelin was continuously administered for five days at doses of 0.09, 0.17, or 0.35 μg/hr. On the same day of the pump implantation, the diet was either continued on NC diet or substituted with LCT or MCT diet. n=5-7/group. [file Image1.tif]

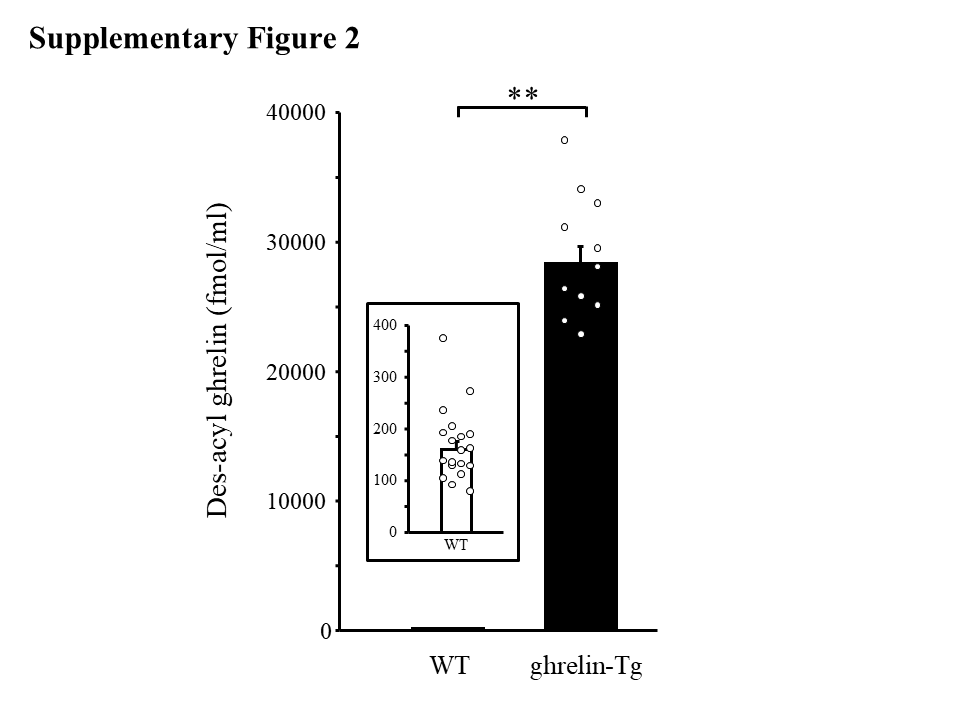

Supplement: Supplementary Figure 2 — Plasma des-acyl ghrelin concentration in ghrelin-Tg and WT mice. Plasma ghrelin concentration was measured in ghrelin-Tg and WT mice fed with NC diet. The inserted panel is magnified view of bar graph of WT mice. n=8/group. **p < 0.01 by non-paired t-tests. [file Image2.tif]
